# In Vitro Study of the Phytochemical Composition and Antioxidant, Immunostimulant, and Hemolytic Activities of *Nigella sativa* (Ranunculaceae) and *Lepidium sativum* Seeds

**DOI:** 10.3390/molecules27185946

**Published:** 2022-09-13

**Authors:** Hafssa Ouattar, Otmane Zouirech, Mohammed Kara, Amine Assouguem, Saeedah Musaed Almutairi, Fahad M. Al-Hemaid, Rabab Ahmed Rasheed, Riaz Ullah, Arshad Mehmood Abbasi, Mahjoub Aouane, Karima Mikou

**Affiliations:** 1 Laboratory of Natural Resources and Sustainable Development, Faculty of Sciences, University of Ibn Tofail, P.O. Box 133, Kenitra 14000, Morocco; 2 Laboratory of Natural Substances, Pharmacology, Environment, Modeling, Health and Quality of Life (SNAMOPEQ), Faculty of Sciences Dhar El Mahraz, University Sidi Mohamed Ben Abdellah, P.O. Box 3000, Fez 30000, Morocco; 3 Laboratory of Biotechnology, Conservation and Valorisation of Naturals Resources (LBCVNR), Faculty of Sciences Dhar El Mehraz, Sidi Mohamed Ben Abdellah University, P.O. Box 1796 Atlas, Fez 30000, Morocco; 4 Laboratory of Functional Ecology and Environment, Faculty of Sciences and Technology, Sidi Mohamed Ben Abdellah University, Imouzzer Street, P.O. Box 2202, Fez 30000, Morocco; 5 Laboratory of Applied Organic Chemistry, Faculty of Sciences and Technology, Sidi Mohamed Ben Abdellah University, Imouzzer Street, P.O. Box 2202, Fez 30000, Morocco; 6 Department of Botany and Microbiology, College of Science, King Saud University, P.O. Box 2455, Riyadh 11451, Saudi Arabia; 7 Histology and Cell Biology Department, Faculty of Medicine, King Salman International University, El Tor 46612, Egypt; 8 Department of Pharmacognosy, College of Pharmacy, King Saud University, P.O. Box 2455, Riyadh 11451, Saudi Arabia; 9 Department of Environmental Sciences, COMSATS University Islamabad, Abbottabad Campus, Abbottabad 22060, Pakistan or; 10 University of Gastronomic Sciences, Piazza Vittorio Emanuele II, 9, 12042 Pollenzo, Italy

**Keywords:** antioxidant activity, hemagglutination, hemolysis, *Lepidium sativum*, medicinal plants, *Nigella sativa*, phytochemistry

## Abstract

The Moroccan flora abounds and is an important reserve of medicinal plants. *Nigella sativa* and *Lepidium sativum* are plants that are widely used in traditional medicine for their multiple therapeutic properties. The current study aims to highlight the biological activities that can justify and valorize the use of these plants. Flavonoids, total phenols, condensed tannins, and sugars were determined. The biological activities tested were antioxidant by determining the IC_50_ (defined as the concentration of an antioxidant required to decrease the initial concentration by 50%; inversely related to the antioxidant capacity), hemagglutination, and hemolytic activities. Phytochemical quantification of the seed extracts indicated that the total phenol content was largely similar for both plants and in the order of 10 mg GAE (Gallic acid equivalent)/g. On the other hand, *L. sativum* seeds registered a higher content of flavonoids (3.09 ± 0.04 mg QE (quercetin equivalent)/g) as compared to *Nigella saliva* (0.258 ± 0.058). Concerning condensed tannins, *N. saliva* seeds present a higher amount with a value of 7.2 ± 0.025 mg/g as compared to *L. sativum* (1.4 ± 0.22 mg/g). Concerning the total sugar content, *L. sativum* shows a higher content (67.86 ± 0.87 mg/g) as compared to *N. sativa* (58.17 ± 0.42 mg/g); it is also richer in mucilage with a content of 240 mg as compared to 8.2 mg for *N. saliva*. Examination of the antioxidant activity using a DPPH (2.2-diphenyl 1-pycrilhydrazyl) test revealed that the EButOH (n-butanol extract) and EAE (ethyl acetate extract) extracts were the most active, with IC_50_ values of 48.7 and 50.65 μg/mL for the *N. sativa* extracts and 15.7 and 52.64 μg/mL for the *L. sativum* extracts, respectively. The results of the hemagglutination activity of the different extracts of the two plants prepared in the PBS (phosphate-buffered saline) medium showed significant agglutination for the *L. sativum* extract (1/50) compared to the *N. sativa* extract (1/20). An evaluation of the hemolytic effect of the crude extract of the studied seeds on erythrocytes isolated from rat blood incubated in PBS buffer compared to the total hemolysis induced by distilled water showed a hemolysis rate of 54% for *Nigella sativa* and 34% for *L. sativum*. In conclusion, the two plants studied in the current work exhibited high antioxidant potential, which could explain their beneficial properties.

## 1. Introduction

Aromatic and medicinal plants are of economic interest in the food, cosmetic, and pharmaceutical industries [1,2]. Indeed, they are endowed not only with aromatic and culinary qualities but also with various medicinal virtues thanks to the different active principles that they contain: alkaloids, flavonoids, tannins, saponosides, and essential oils [3,4,5,6,7]. They constitute an inexhaustible reservoir of the most effective folk remedies and are the most widely used natural source of medicines today [8,9,10].

In Morocco, bioclimatic diversity is one of the reasons for the richness and variousness of its flora, particularly in terms of medicinal and aromatic plants (MAP). Medicinal flora is widely used by the Moroccan population [9,10,11]. Indeed, most rural populations suggested that MAP occupy an important place in the treatment strategies of various ailments as natural remedies [12].

*Nigella sativa* L. (Ranunculaceae) is an annual plant with an erect, ribbed, angular, and twiggy stem that is 60 cm high. It is one of the medicinal plants that occupy a special place in the world of traditional medicine because of its wide therapeutic uses by the populations of Arab countries, Asia, and Europe against numerous pathologies, such as hypertension, diabetes, fever, migraine, inflammation, and gastrointestinal problems [13,14]. In addition, *Lepidium sativum* L. (Brassicaceae) is an annual plant that originated in Egypt and western Asia but is now cultivated throughout the world. Its seeds are used fresh or dried to treat sore throats, coughs, asthma, headaches, and stomach aches [15].

Oxidative stress is one of the most abundant problems in the biological and medical world. It is a situation in which a cell can no longer resist the exhaustive production of toxic free radicals, which leads to many dangerous diseases, such as cancer [16,17]. ROS are always present in our bodies, as oxidation is part of the aerobic life of our metabolism, but they all have limits, as overproduction of these species can be harmful to the body [18,19].

Traditional formulations have constituted the first line of healthcare of different civilizations since time immemorial. Researchers have focused on the phytochemical composition of plants to explain their beneficial properties. Within this scope, we testify to the antioxidant, hemagglutination, and hemolytic activities of two medicinal plants (*N. sativa* and *L. sativum*).

The approach taken in this study, which is devoted to the study of the seeds of medicinal plants (*Nigella sativa* and *Lepidium sativum*), consists of the determination of the chemical compounds of the seed extracts. Among these compounds, those with antioxidant activity (phenolic compounds, flavonoids, condensed tannins, and fixed oils) are qualified as a secondary metabolites extract.

## 2. Materials and Methods

### 2.1. Extract Preparation

Seeds of *Nigella sativa* and *Lepidium sativum* were ground using an electric grinder to obtain a fine powder. The plants were collected in the Souk El Arbaa area of Morocco 5°58′54″ W 34°39′57″ N. An amount of 1 g of plant powder was mixed with 10 mL of methanol/water (*v*/*v*) under agitation overnight at room temperature. The extract was then centrifuged at 5000 rpm for 5 min. The supernatant was recovered to perform different dosages. Fractionation was carried out using the liquid/liquid method with ethyl acetate and methanol solvent.

### 2.2. Chemical Reagents

Folin–Ciocalteu, ascorbic acid, gallic acid, AlCl_3_ (aluminum trichloride), quercetin, DPPH (1,1-diphenyl-2-picryl-hydrazyl), NaCl, KCl, NA_2_HPO_4_, Na_2_CO_3_, KH_2_PO_4_, concanavalin, methanol, chloroform, and ethanol were used as solvents.

### 2.3. Determination of Total Phenols

A volume of 0.5 mL of the diluted extract was added to 3 mL of distilled water and 0.5 mL of 20% Na_2_CO_3_. The mixture was vortexed and 0.5 mL of Folin–Ciocalteu reagent was added after 3 min. The tubes were placed in an incubator at 40 °C for 30 min. The absorbance was subsequently read at 760 nm [20].

The results were expressed as mg of gallic acid per g of seed extract (mg GAE/g).

### 2.4. Determination of Condensed Tannins

The technique used is based on the ability of tannins to be transformed into anthocyanins by heating in an acid medium [21]. Two series of tubes were prepared. In each series, 3 mL of the ethanolic extract was added to 3 mL of concentrated HCl. Series 1 tubes were placed in a water bath at 100 °C for 30 min, followed by rapid cooling under a jet of water. Series 2 tubes were kept at room temperature. In both series, 0.5 mL of concentrated ethanol was added. The absorbance was read using a spectrophotometer at 550 nm, and the condensed tannin content was determined using the following formula:

CTC = (OD1 − OD2) × 19.33 (g/L)

where CTC represents the condensed tannin content, OD1 represents the optical density of the series 1 tubes, and OD2 represents the optical density of the series 2 tubes.

### 2.5. Determination of Flavonoids

The aluminum trichloride (AlCl_3_) method is used to quantify the flavonoids of the seed extracts [22]. Briefly, 2 mL of AlCl_3_ solution at 20 g/L was added to 2 mL of sample or standard (prepared in methanol). The mixture was incubated in the dark for 15 min. The blank was made by replacing the extract with methanol; the absorbance was recorded at 425 nm, and the results were expressed as mg of quercetin per g of seed extract (mg QE/g) [23].

### 2.6. Determination of Total Sugars

The determination of the total sugars was carried out by the phenol-sulfuric method. In a hot sulfuric solution, the carbohydrates are dehydrated to furfural derivatives, which combine readily with phenol to give a yellow-brown complex (glucose provides hydroxyfurfural).

The quantification of the total sugars was carried out by an external calibration range prepared from a glucose stock solution and spreading from 5 µg/mL to 100 µg/mL.

### 2.7. Quantification of Mucilage

The seeds were macerated in distilled water (*w*/*v*) at ambient temperature. The mixture was then brought to a boil. After cooling, the mixture was filtered to remove insoluble macroscopic debris [24]. Acetone was added to the filtrate to precipitate the mucilage. After centrifugation, the mucilage was separated from the supernatant and oven-dried at 45 °C. They were then collected as a powder and weighed to calculate the yield as follows:

Yield = (m/m′) × 100

where m represents the mass (in g) of the powder and m’ represents the mass (in g) of the test portion of the plant drug.

### 2.8. Extraction of Fixed Oils

The extraction of the fixed oils is realized according to the protocol described by Ramadan et al. [25] with modifications. An amount of 20 g of seed powder was mixed with 100 mL of chloroform. After 2 h, the mixture was then filtered. The chloroform was eliminated by evaporation under reduced pressure at 40 °C using a Rota vapor (BÜCHI). This operation produces an extract characterized by a dark green color, which is considered to be the chloroform extract of fixed oils.

The yield of the fixed oil extraction is expressed as a percentage of the weight of the seeds and calculated according to the following formula:

Fixed oil (%) = (Weight of oil/weight of seeds) × 100

### 2.9. Biological Activities

#### 2.9.1. Antioxidant Activity: DPPH Test

The protocol used is that described by Sharma and Bhat [26]. An amount of 2.5 mL of different concentrations of the extracts is mixed with 2.5 mL of a DPPH solution (100 μM) prepared in methanol. The reaction mixture is stirred immediately and then maintained in the dark for 30 min at ambient temperature (25 °C). The absorbance was measured at 517 nm against a blank containing only methanol. The control consisted of a reaction mixture containing 2.5 mL DPPH and 2.5 mL methanol. The same test was carried out with a reference antioxidant, which is ascorbic acid. The measurements of the absorbance of DPPH of the different antioxidant substances (extracts and ascorbic acid) allow the determination of the percentage of inhibition (PI) by applying the following formula [27,28]:

PI (%) = (1 − (Abs test/Abs control)) × 100

IC_50_ represents the concentration of the substance (extract or ascorbic acid) necessary to decrease the free radicals in the reaction medium by 50% [29]. IC_50_ values are calculated by linear regression in which the abscissa is represented by the concentration of the tested compounds, and the ordinate is represented by the percentage of inhibition (PI%).

#### 2.9.2. Phyto-Hemagglutination Activity

This work was carried out on red blood cells from rat blood. The test is based on the observation of the agglutination or precipitation of erythrocytes in the presence of extracts from our plants, which can confirm or deny the presence of lectins. The hemagglutination activity is then demonstrated when there is the precipitation of erythrocytes.

##### Preparation of the Total Extract

This operation consists of extracting water-soluble substances from the seed powder using a phosphate-buffered saline (PBS). An amount of 20 g of powder was macerated in 200 mL of PBS for 24 h. The mixture was left under agitation for 2 h. After centrifugation of this suspension at 3000 rpm for 15 min, the supernatant was recovered and conserved at 4 °C. The supernatant obtained constitutes the total extract. This extract is kept and used for hemagglutination and hemolysis tests.

##### Red Blood Cell Preparation

The rat blood was collected in a heparinized test tube to stop coagulation. It was centrifuged at 5000 rpm for 3 min. The supernatant was eliminated, and then a physiological solution (0.9% NaCl) was added to the erythrocyte pool. This lavage operation was repeated four times under the same conditions. After the fourth wash, a 3% red cell solution was prepared in 0.93% NaCl.

##### Hemagglutination Test

This test is based on the observation of the agglutination of red blood cells and the consequent precipitation of erythrocytes [30]. In the present study, rat red blood cells were used. It was performed in 96-well round-bottom plates. For this, in each well, 50 μL of 0.9% NaCl, 25 μL of the test extract, and 25 μL of the red blood cells (3%) were deposited. The plate was then covered and incubated in an oven at 37 °C for 30 min before reading the results. Two controls were used; the extract was replaced by concanavalin (positive control) or 0.9% NaCl (negative control).

##### The Hemagglutination Limit

The objective of this test is to determine the minimum concentration of the extract that causes agglutination of erythrocytes. For this purpose, a range of dilutions of each extract from 1/5 to 1/2000 times was performed. The hemagglutinating activity was determined after 1 h of incubation at room temperature. The observation was performed with the naked eye and by light microscope (G × 40).

#### 2.9.3. Hemolytic Activity

Plants are also known for their toxic effects, which led us to study the hemolytic effect, in vitro, of these two plants (*Nigella sativa* and *Lepidium sativum*). The test of the hemolytic effect of the two plants was carried out on the crude extract (PBS) previously prepared and also used for the test of the phyto-hemagglutination activity. The test for the hemolytic effect of the studied plant is carried out according to the methods described by [31].

In hemolysis tubes, 2970 μL of the prepared erythrocyte suspension was mixed with 30 μL of the extract at different initial concentrations (10 mg/mL, 25 mg/mL, 50 mg/mL, and 100 mg/mL). The individual tubes were incubated at 37 °C for one hour. Every 15 min for 60 min, 500 μL of each tube was taken and mixed with 1.5 mL of PBS. The individual tubes were centrifuged at 3000 rpm for 10 min; the pellet was removed, and the absorbance of each tube was read at 548 nm (the absorbance of hemoglobin) using a UV-visible spectrophotometer against a blank containing PBS. A negative control tube was prepared under the same experimental conditions. It consisted of 500 μL of erythrocyte suspension and 1500 μL of PBS buffer, in the absence of extract.

##### Rate of Hemolysis

Under the same conditions, a total hemolysis tube was also prepared; this tube contained 500 μL of the erythrocyte suspension and 2500 μL of distilled water. The rate of hemolysis of the different extracts was calculated as a percentage (%) of the total hemolysis after 60 min of incubation according to the formula [32]:

Hemolysis rate (%) = (Absorbance of extract in 1H − Absorbance of negative test at 1H/Total hemolysis after 1H).

### 2.10. Statistical Analysis

The results of the analyses performed in Excel are expressed as mean ± standard error. The Tukey post-hoc multiple comparison of means test was used to test for significant differences between the means (at the 5% level). These statistical analyses were performed with IBM.SPSS, Version 19. The results of each experiment were completed in triplicate.

## 3. Results

### 3.1. Results of Humidity, Sugar Content, and Mucilage

The obtained results for the humidity, sugar content, and mucilage are presented in Table 1. The data revealed that the humidity rate for the seeds is lower than 10%, and the proportions of both seed extracts are 8.40 ± 0.03% and 9.54 ± 0.18% for *N. sativa* and *L. sativum*, respectively. Interestingly, the water content plays an important role in the seeds’ conservation; values lower than 10% give seeds better long-term conservation.

The results of the sugar contents of the *Nigella sativa* and *Lepidium sativum* seeds are depicted in Table 1. The highest value was recorded in *L. sativum* with a tenor of about 67.86 ± 0.87 mg/g, while *N. sativa* contains the lowest amount of sugars with a value of (58.17 ± 0.42 mg/g).

Concerning the mucilage quantification, the results showed that the extract of *L. sativum* scored a high value of mucilage, while *N. sativa* contained the lowest amount of mucilage with a value of 8.2 mg/g.

### 3.2. Phytochemical Quantification of N. sativa and L. sativum Seeds

The concentration of total polyphenols is determined using the Folin–Ciocalteu method with gallic acid as the standard. The findings were expressed in mg gallic acid equivalent per gram of dry matter (mg GAE/g). The results are summarized in Table 2. The results of the polyphenol quantification showed that the crude methanolic extract of *Nigella sativa* registered an amount of TPC of 10.48 ± 1.33 mg EAG/g. *Lepidium sativum* recorded a value of 10.35 ± 0.73 mg EAG/g. In the same context, the flavonoid content quantification showed that the lowest content registered in the *N. sativa* extract, while the other vegetal matrix contained a high amount of TFC (3.09 ± 0.048 mg QE/g).

Concerning the condensed tannins, the amounts obtained ranged from 1.4 ± 0.22 to 7.2 ± 0.025 mg/g. *N. sativa* appeared to be the herb richest in condensed tannins, with an amount of 7.2 ± 0.025 mg/g. In contrast, *L. sativum* registered the lowest content of condensed tannins with a value of 1.4 ± 0.22 mg/g.

### 3.3. Antioxidant Activity

#### 3.3.1. For Extracts and Fractions

**Figure 1:**
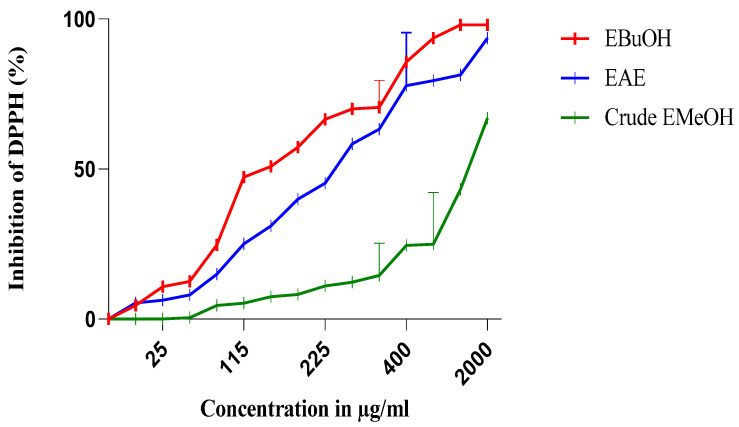
Inhibition of DPPH (%) by the n-butanol (EBuOH), ethyl acetate (EAE), and crude methanolic extract (crude EMeOH) fractions of *N. sativa* seeds.

**Figure 2:**
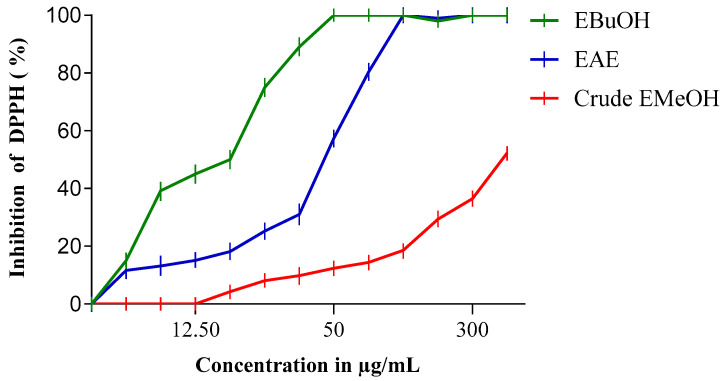
Inhibition of DPPH (%) by n-Butanol (EBuOH), ethyl acetate (EAE), and crude methanol extract (Crude EMeOH) fractions of *L. sativum* Seeds.

The antiradical activity profiles obtained are represented in Figure 1 and Figure 2. The extracts of *N. sativa* (Figure 1) have a dose-dependent antiradical activity. The crude extract has an activity lower than that of the n-butanol fraction (EButOH), which represents the most active fraction with an IC50 of the order of 48.7 μg/mL, followed by the ethyl acetate (EAE) fraction with an IC_50_ of 50.65 μg/mL. The study of the antioxidant activity of extracts from the seeds of *L. sativum* (Figure 2) also reveals the significant activity of the n-butanol fraction and the ethyl acetate fraction with respective IC_50_ values of 15.7 and 52.64 μg/mL.

The antioxidant activity of the methanolic extracts of the two studied seeds and their fractions was evaluated by the percentage of DPPH radical inhibition, and the obtained results are depicted in Table 3.

The methanolic extract (crude EMetOH) of *Nigella sativa* seeds converts the stable free radical (2.2 diphenyl-1-picrylhydrazyl) into the yellow-colored diphenyl-picrylhydrazine with an IC_50_ value in the order of 1331.53 μg/mL, showing an activity that is practically inferior to that of *Lepidium sativum*, which renders stability to DPPH with an IC_50_ of 380 μg/mL.

Concerning the different fractions carried out, we tried to compare different extracts according to their capacities to scavenge DPPH• and thus appreciate the qualitative variations of the phenolic compounds, in order to establish a relationship between the contents of the phenolic compounds and the antioxidant activity of the different fractions [33]. It should be noted that these solvents are used for their selective power. According to [34], ethyl acetate is used to extract mono-, di-, and tri-O-glycoside flavonoids; n-butanol is used for the extraction of di-o-glycoside, tri-glycoside, and C-glycoside flavonoids; chloroform is used for the extraction of the organic phase containing aglycone and methoxylated aglycone flavonoids; and hexane is used for the de-icing of the extract.

The extraction was carried out with methanol 85%. The use of methanol with water at a proportion of 15% can lead to the co-extraction of contaminants, such as wax, lipids, and chlorophylls [19]. Therefore, fractionation of the obtained extracts was carried out using solvents of increasing polarity.

Among the two fractions of *Nigella sativa*, the n-butanol fraction (EButOH) represents the most active fraction with an IC_50_ of about 48.7 μg/mL, followed by EAE with an IC_50_ of 50.65 μg/mL (see Table 3).

The study of the antioxidant activity of the extracts from *Lepidium sativum* (Table 3) showed that the n-butanol fraction (EButOH) and the ethyl acetate fraction (EAE) represent the two most active fractions among the three fractions tested in this study, with IC_50_ values of 15.7 and 52.64 μg/mL, respectively.

#### 3.3.2. For Vegetal Oils

**Figure 3:**
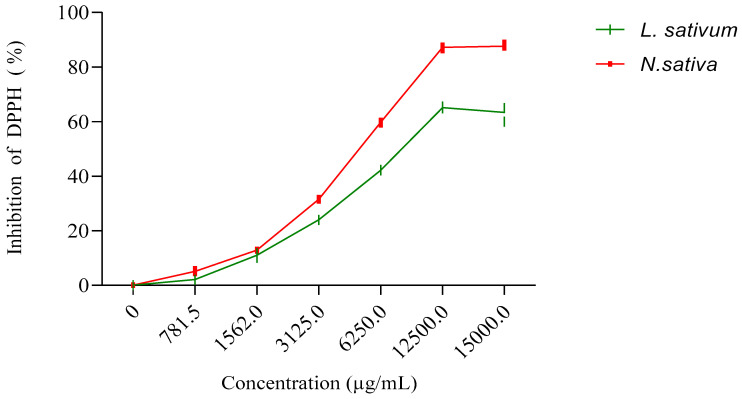
Inhibition of DPPH (%) by fixed oils extracted from seeds of *N. sativa* and *L. sativum*.

The results obtained (Figure 3) show that the fixed oil extracted from the seeds studied has an antioxidant activity that is greater for the oil of *N. sativa*, with an IC50 of 6374 μg/mL, than for the oil extracted from *L. sativum*, with an IC50 of 9005.54 μg/mL. However, this activity of the oils remains much lower than those recorded for the methanolic extracts and the n-butanol and ethyl acetate fractions (Table 3).

**Figure 4:**
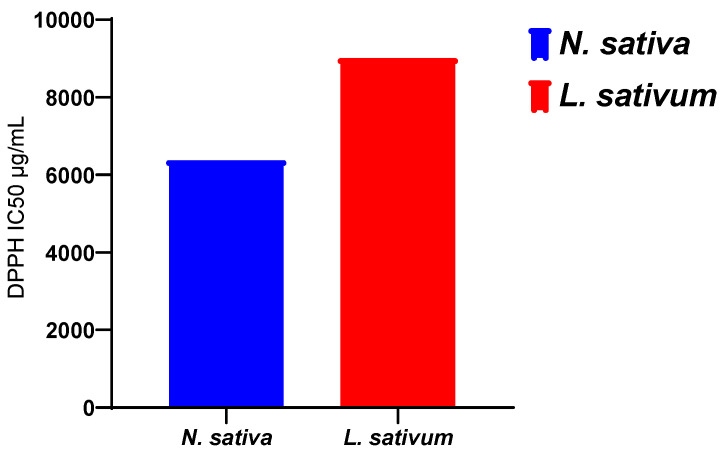
The scavenging free radical activity of vegetal of seeds of *N. sativa* and *L. sativum*.

Figure 1 displays the results obtained for the antioxidant activity of the vegetal oils of the seeds of both studied plants. The obtained results revealed that the vegetal oil of *Nigella sativa* exhibited higher antioxidant activity, with an IC_50_ of 6374 μg/mL, compared to *Lepidium sativum* seeds, with registered an IC_50_ of 9005.54 μg/mL (Figure 4).

### 3.4. Hemagglutination Activity

#### 3.4.1. Phyto-Hemagglutination Test

**Figure 5:**
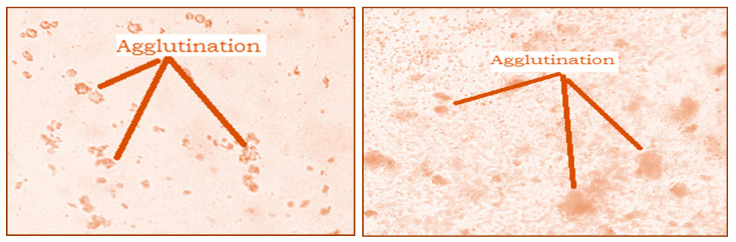
Microscopic observation (G × 40) of agglutination of rat red blood cells by *Nigella sativa* and *Lepidium sativum* extracts.

The hemagglutination activity of the studied extracts was presented in Figure 5. The analysis of the obtained results showed a significant agglutination of red blood cells after 30 min of incubation. This agglutination is observed both with the naked eye and under the microscope (Figure 5). This result shows that both extracts contain lectins, which interact with red blood cells to form a homogeneous cluster in the form of a gelatinous phase.

#### 3.4.2. Hemagglutination Limit Test

The hemagglutination limit test is used to assess the minimum concentration that induces hemagglutination activity. The hemagglutination activity is expressed as a titer, which is the reciprocal of the highest dilution ratio at which hemagglutination is observed.

**Figure 6:**
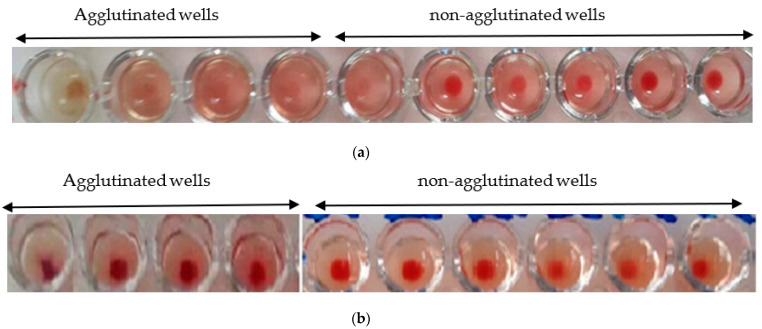
(**a**) Hemagglutination limit of *Nigella sativa*: observation with the naked eye; (**b**) hemagglutination limit of *Lepidium sativum*: observation with the naked eye.

The minimum hemagglutination activity of the *Nigella sativa* extract is 1/20 (Figure 6a), while, for the *L. sativum*, it is 1/50 (Figure 6b).

### 3.5. Evolution of the Hemolytic Effect of Seeds

The hemolysis test is an indicator of cytotoxicity based on the hemolysis of rat red blood cells at different concentrations of the crude extracts of the *Nigella sativa* and *Lepidium sativum* seeds. This parameter is widely used to evaluate the antioxidant ability of extracts, which helps in phytotherapy and pharmacological preparations.

The obtained results show an increase in absorbance throughout the experience time at 0, 15, 30, and 60 min in proportion to the concentrations of *Nigella sativa* and *Lepidium sativum* extracts. This result reflects an increase in the rate of hemolysis with an increasing extract concentration.

**Figure 7:**
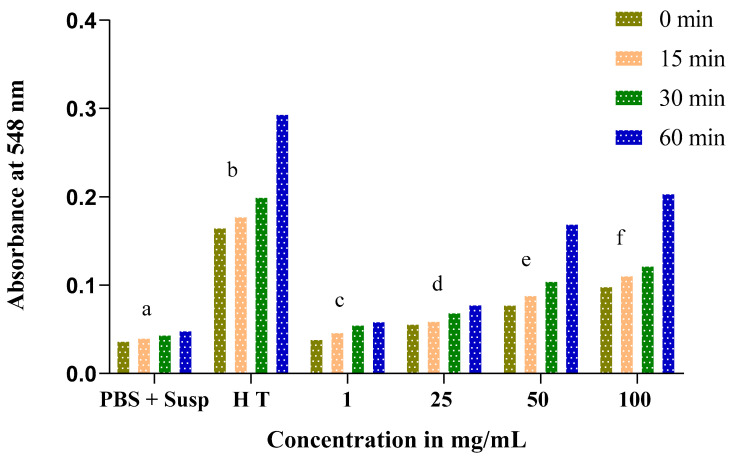
Time course of absorbance of tubes containing an erythrocyte suspension in the presence of different concentrations of total *Nigella sativa* extract incubated at 37 °C compared to total hemolysis (H T) and negative control (PBS + Susp.). The same letters (a, b, c, d, e, f) denote a significant difference with respect to the absorbance.

At low concentrations of both the *Nigella sativa* and *Lepidium sativum* extracts (1 and 25 mg/mL), the absorbance rate does not exceed 0.1. At a dose of 100 mg/mL, we recorded a remarkable hemolytic effect of the total extract of the *Nigella sativa* seeds. In addition, a significant increase in absorbance was remarked from 0.098 at 0 min to 0.204 at 60 min (Figure 7).

**Figure 8:**
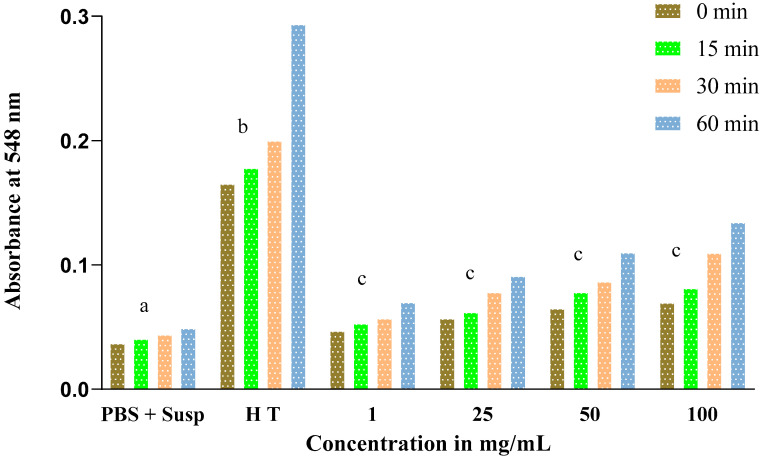
Evolution of absorbance in tubes containing erythrocyte suspension as a function of different concentrations of the total extract of Lepidium sativum. Letters (c) indicate a non-significant difference. Different letters (a,b) indicate a significant difference.

Similarly, for *Lepidium sativum*, we noted that the absorbance also increases depending on the concentration (Figure 8) but does not exceed a value of 0.14 for the final concentration of 100 mg/mL at 60 min of incubation. This increase is not significant.

### 3.6. Estimation of Hemolysis Rate

**Figure 9:**
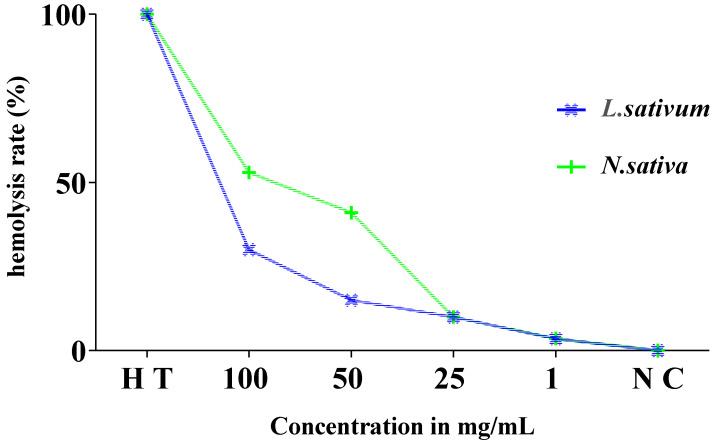
Evolution of hemolysis rate (%) of red blood cells in the presence of different concentrations of *Nigella sativa* and *Lepidium sativum* seed extract after 60 min incubation compared to total hemolysis (H T) and the negative control (T N).

Figure 9 displays the results of the estimation of the hemolysis rate. The analysis of the findings revealed very low rates of hemolysis of the order of 3.6%, 10%, and 41% in relation to the total hemolysis noted for the concentrations of 1, 25, and 50 mg/mL, respectively. This rate is moderately higher, by 53%, at a concentration of 100 mg/mL of the studied extract compared to the positive control (total hemolysis).

The hemolytic effect of the total extract of the *Lepidium sativum* seeds (Figure 9) showed that these seeds present a very weak toxic effect on isolated erythrocytes, with a rate of hemolysis that does not exceed 15% at a concentration of 50 mg/mL compared to the total hemolysis. It is 30% for a concentration of 100 mg/mL.

## 4. Discussion

The quantification of the polyphenolic content of *Nigella sativa* shows lower amounts than those found previously by [33]. For *Lepidium sativum,* the results obtained showed higher amounts of phenolic compounds as compared to those obtained by (8.651 ± 0.321 mgEAG/g) [35]. It should be noted that the phenolic content of plants is highly linked to numerous intrinsic and extrinsic factors, such as geographical origin, climatic conditions, cultivation practices, maturity at harvest, and storage conditions [36].

A considerable flavonoid amount is unregistered in the methanolic extract of *Nigella sativa*. Our findings are in accordance with those evoked by [37]. Concerning *Lepidium sativum*, the quantification of TPC revealed that our plant contained lower amounts than those reported by (4.023 ± 0.081 mgEC/g) [38].

*Nigella sativa* seed extract contains a large number of bioactive compounds, such as flavonoids, phenolics, alkaloids, saponins, glycoside, coumarins, fixed oils, proteins, vitamins, and minerals [39]. The same ascertainment is found for *L. sativum* with a high mucilage proportion; based on this feature, *L. sativum* mucilage was used as a natural polymer that is facilely disintegrated, non-toxic, and biodegradable, providing rapid release of an encapsulated drug [40]. Furthermore, it has been suggested that supplementation of the *N. sativa* extract at a dose of 16% in a broiler diet improves different blood parameters viz. humoral hormone response, serum profile markers, and significant changes in the hemogram and leukogram profiles with non-side effects remarked [41]. In fact, medicinal plants as multipurpose products act on different biological systems to provide their pharmacological properties [42,43,44,45,46]. The bioactive compounds provide an important force to attenuate free radicals induced by numerous toxic agents, such as hydrogen peroxide and gentamicin [42,47]. The fractionation technique is used broadly to recuperate targeted components with a high added value as effective antioxidant compounds [48]. Our findings revealed a good ability to scavenge free radical DPPH from crude extracts (Table 3). Previously, Jallol et al. proved that the fractions recorded a high amount of phenolic compounds and the highest antioxidant ability [49], which is in line with our findings. The antioxidant activity of a plant extract depends on the composition and structure of biocompounds, such as phenolic acids and flavonoids, and their ability to neutralize reactive oxygen species (ROS) and other free radicals, i.e., chelating and free radical scavenging activities.

Concerning the agglutination test, both of the plant extracts exhibited an important agglutination effect, and our results are in concordance with those reported by [50,51]. Phytolectins are the components most known for their ability to attach erythrocytes, thereby forming a network of erythrocytes that results in hemagglutination [51]. Phytolectins are implicated in different biomedical research fields as antifungal, anti-inflammatory, anticancer, and antibacterial agents [52]. In light of the results obtained from the hemolytic tests carried out in vitro on the erythrocytes isolated from rat blood, the seeds of both plants are low in toxicity. They can be very important sources in the therapeutic and pharmacological fields to alleviate various diseases.

The interest of our study is obvious: firstly, in case of validation of the antioxidant hemagglutinin and antihemolytic properties of the plants, the traditional knowledge and modern technology of pharmacology and chemistry will be integrated in order to develop effective and non-toxic phytomedicines to fight against several health problems that aggravate poverty in North Africa.

## 5. Conclusions

This study shows that the seeds of the studied plants are more or less rich in metabolites with significant therapeutic activity, which gives these plants interesting biological activities and thus can justify their traditional uses. The difference thus observed in the biological activity of these two seeds of the studied plants may be due to the quality and/or quantity of the extracts in bioactive secondary metabolites. The methanolic fractions exhibited are an effective scavenger of free radicals and, therefore, a powerful inhibitor of DPPH due to the high yield of phenolic compounds. The hemagglutination test with the total extract of *Nigella sativa* shows a relatively higher agglutinating effect than the extract of *Lepidium sativum.* The seeds of both plants are weakly toxic. They can be a very important source in the therapeutic and pharmacological fields.

## Figures and Tables

**Table 1:** Humidity, sugar content, and mucilage of both seed extracts.

	Humidity %	Sugar Content (mg/g)	Mucilage (mg/g)
*N. sativa* seeds	8.40 ± 0.03	58.17 ± 0.42	8.2
*L. sativum* seeds	9.54 ± 0.18	67.86 ± 0.87	240

**Table 2:** Phytochemical content of both seed extracts.

	TPC mg GAE/g	TFC mg QE/g	CT mg/g
*N. sativa* seeds	10.48 ± 1.33	0.258 ± 0.058	7.2 ± 0.025
*L. sativum* seeds	10.35 ± 0.73	3.09 ± 0.048	1.4 ± 0.22

TPC: total polyphenols, TFC: flavonoid content, and CT: tannin content.

**Table 3:** The IC50% of different extracts of *N. sativa* and *L. sativum*.

	*Nigella sativa*			*Lepidium sativum*		
	Crude Extract EMetOH	Fraction		Crude Extract EMetOH	Fraction	
		EButOH	EAE		EButOH	EAE
DPPH IC_50_ (µg/mL)	1331.53	48.7	50.65	380	15.7	52.64

EButOH: n-butanol fraction, EAE: ethyl acetate fraction, EMetOH: methanolic extract.

## Data Availability

Not applicable.

## References

[1] Oulidi J., Lamiaceae L. (2015). Étude ethnobotanique des plantes médicinales et aromatiques utilisées dans la ville de Fès au Maroc—Ethnobotanical survey of medicinal and aromatic plants used by the people of Fez in Morocco. Phytothérapie.

[2] Saad B., Azaizeh H., Abu-hijleh G., Said O. (2006). Safety of traditional Arab herbal medicine. Evid.-Based Compl. Altern. Med..

[3] Proestos C., Boziaris I.S., Nychas G.E., Komaitis M. (2006). Food chemistry analysis of flavonoids and phenolic acids in Greek aromatic plants: Investigation of their antioxidant capacity and antimicrobial activity. Food Chem..

[4] Benmakhlouf Z., Benserradj O., Kellab R. (2022). Short communication: Identification of phytochemical constituents of Syzygium Aromaticum L. Using gas chromatography coupled with mass spectrometry and evaluation of antimicrobial activity. Biodiversitas J. Biol. Divers..

[5] Ullah R., Alqahtani A.S. (2022). GC-MS Analysis, Heavy Metals, Biological, and Toxicological Evaluation of Reseda muricata and Marrubium vulgare Methanol Extracts. Evidence-Based Complement. Altern. Med..

[6] Bouslamti M., El Barnossi A., Kara M., Alotaibi B.S., Al Kamaly O., Assouguem A., Lyoussi B., Benjelloun A.S. (2022). Total polyphenols content, antioxidant and antimicrobial activities of leaves of Solanum elaeagnifolium Cav. from Morocco. Molecules.

[7] Assouguem A., Kara M., Ramzi A., Annemer S., Kowalczyk A., Ali E.A., Moharram B.A., Lazraq A., Farah A. (2022). Evaluation of the effect of four bioactive compounds in combination with chemical product against two spider mites Tetranychus urticae and Eutetranychus orientalis (Acari: Tetranychidae). Evid.-Based Compl. Altern. Med..

[8] Salhi S., Fadli M., Zidane L., Douira A. (2011). Etudes floristique et ethnobotanique des plantes medicinales de la ville de Kénitra (Maroc). REVESCO Rev. Estud. Coop..

[9] El Khomsi M., Kara M., Hmamou A., Assouguem A., Al Kamaly O., Saleh A., Ercisli S., Fidan H., Hmouni D. (2022). In vitro studies on the antimicrobial and antioxidant activities of total polyphenol content of Cynara humilis from Moulay Yacoub area (Morocco). Plants.

[10] Hmamou A., Eloutassi N., Alshawwa S.Z., Al kamaly O., Kara M., Bendaoud A., El-Assri E.-M., Tlemcani S., El Khomsi M., Lahkimi A. (2022). Total phenolic content and antioxidant and antimicrobial activities of Papaver rhoeas L. organ extracts growing in Taounate Region, Morocco. Molecules.

[11] Assouguem A., Kara M., Mechchate H., Al-Mekhlafi F.A., Nasr F., Farah A., Lazraq A. (2022). Evaluation of the Impact of Different Management Methods on Tetranychus urticae (Acari: Tetranychidae) and Their Predators in Citrus Orchards. Plants.

[12] Ali B.H., Blunden G. (2003). Pharmacological and toxicological properties of nigella sativa. Phytother. Res. Int. J. Devot. Pharmacol. Toxicol. Eval. Nat. Prod. Deriv..

[13] Ennabili A., Gharnit N., Maach Y., El Meskaoui A., Bousta D. (2006). Exploitation des plantes medicinales et al.imentaires du bassin versant de l’oued laou (Nord-Ouest du Maroc). J Bot. Soc. Bot. Fr..

[14] Bnouham M., Mekhfi H., Legssyer A., Ziyyat A. (2002). Ethnopharmacology forum medicinal plants used in the treatment of diabetes in Morocco. Int. J. Diabetes Metab..

[15] Favier A. (2003). Le Stress Oxydant Intérêt Conceptuel et Expérimental dans la Compréhension des Mécanismes des Maladies et Potentiel Thérapeutique. L’actualité Chim..

[16] Favier A. (2006). Stress oxydant et pathologies humaines. Ann. Pharm. Fr..

[17] Nath K.A., Norby S.M. (2000). Reactive oxygen species and acute renal failure of reactive oxygen species. Am. J. Med..

[18] Bandyopadhyay U., Das D., Banerjee R.K. (1999). Reactive oxygen species: Oxidative da and pathogenesis. Curr. Sci..

[19] Mompon B., Lemaire B., Mengal P., Surbled M. (1998). Extraction des polyphenols: Du laboratoire a la production industrielle. Colloq.-INRA.

[20] Chung K.-T., Wong T.Y., Wei C.-I., Huang Y.-W., Lin Y. (1998). Tannins and human health: A review. Crit. Rev. Food Sci. Nutr..

[21] Bahorun T., Gressier B., Trotin F., Brunet C., Dine T., Luyckx M., Vasseur J., Cazin M., Cazin J.C., Pinkas M. (1996). Oxygen species scavenging activity of phenolic extracts from hawthorn fresh plant organs and pharmaceutical preparations. Arzneimittelforschung.

[22] Kara M., Assouguem A., Abdou R.Z., Bahhou J. (2021). Phytochemical content and antioxidant activity of vinegar prepared from four apple varieties by different methods. Trop. J. Nat. Prod. Res..

[23] Monrroy M., García E., Ríos K., García J.R. (2017). Extraction and physicochemical characterization of mucilage from *Opuntia cochenillifera* (L.) Miller. J. Chem..

[24] Ramadan M.F., Kroh L.W., Mörsel J.-T. (2003). Radical scavenging activity of Black Cumin (*Nigella sativa* L.), Coriander (*Coriandrum Sativum* L.), and Niger (*Guizotia abyssinica* Cass.) crude seed oils and oil fractions. J. Agric. Food Chem..

[25] Sharma O.P., Bhat T.K. (2009). DPPH antioxidant assay revisited. Food Chem..

[26] Mensor L.L., Menezes F.S., Leitão G.G., Reis A.S., dos Santos T.C., Coube C.S., Leitão S.G. (2001). Screening of Brazilian plant extracts for antioxidant activity by the use of DPPH free radical method. Phytother. Res..

[27] Kara M., Assouguem A., Fadili M.E., Benmessaoud S., Alshawwa S.Z., Kamaly O.A., Saghrouchni H., Zerhouni A.R., Bahhou J. (2022). Contribution to the evaluation of physicochemical properties, total phenolic content, antioxidant potential, and antimicrobial activity of vinegar commercialized in Morocco. Molecules.

[28] Kähkönen M.P., Hopia A.I., Vuorela H.J., Rauha J.-P., Pihlaja K., Kujala T.S., Heinonen M. (1999). Antioxidant activity of plant extracts containing phenolic compounds. J. Agric. Food Chem..

[29] Carlini C.R., Udedibie A.B. (1997). Comparative effects of processing methods on hemagglutinating and antitryptic activities of canavalia ensiformis and canavalia Braziliensis seeds. J. Agric. Food Chem..

[30] Sharma P., Sharma J.D. (2001). In vitro hemolysis of human erythrocytes—By plant extracts with antiplasmodial activity. J. Ethnopharmacol..

[31] Ktari N., Trabelsi I., Bardaa S., Triki M., Bkhairia I., Salem R.B.S.-B., Nasri M., Salah R.B. (2017). Antioxidant and hemolytic activities, and effects in rat cutaneous wound healing of a novel polysaccharide from Fenugreek (*Trigonella foenum-graecum*) Seeds. Int. J. Biol. Macromol..

[32] Meziti A., Meziti H., Boudiaf K., Mustapha B., Bouriche H. (2012). Polyphenolic profile and antioxidant activities of Nigella Sativa seed extracts in vitro and in vivo. Int. J. Biotechnol. Bioeng..

[33] Asdadi A., Harhar H., Gharby S., Bouzoubaâ Z., Yadini A.E., Moutaj R., Hadek M.E., Chebli B., Hassani L.M.I. (2014). Chemical composition and antifungal activity of Nigella Sativa L. oil seed cultivated in Morocco. Int. J. Pharm. Sci. Invent..

[34] Al-Snafi A.E. (2020). Chemical constituents and pharmacological effects of melilotus officinalis—A review. IOSR J. Pharm..

[35] Oracz J., Zyzelewicz D., Nebesny E. (2015). The content of polyphenolic compounds in cocoa beans (*Theobroma cacao* L.), depending on variety, growing region, and processing operations: A review. Crit. Rev. Food Sci. Nutr..

[36] Lefahal M., Zaabat N., Ayad R., Makhloufi E.H., Djarri L., Benahmed M., Laouer H., Nieto G., Akkal S. (2018). In vitro assessment of total phenolic and flavonoid contents, antioxidant and photoprotective activities of crude methanolic extract of aerial parts of *Capnophyllum peregrinum* (L.) Lange (Apiaceae) growing in Algeria. Medicines.

[37] Doke S., Guha M. (2014). Garden Cress (*Lepidium sativum* L.) seed—An important medicinal source: A. Cellulose.

[38] Dhaheri Y.A., Wali A.F., Akbar I., Rasool S., Razmpoor M., Jabnoun S., Rashid S., Khan A., Rehman M. (2022). Chapter 3—Nigella Sativa, a Cure for Every Disease: Phytochemistry, Biological Activities, and Clinical Trials. Black Seeds (Nigella Sativa).

[39] Alam M.T., Parvez N., Sharma P.K. (2014). FDA-approved natural polymers for fast dissolving tablets. J. Pharm..

[40] Talebi A., Maham M., Asri-Rezaei S., Pournaghi P., Khorrami M.-S., Derakhshan A. (2021). Effects of Nigella Sativa on performance, blood profiles, and antibody titer against newcastle disease in broilers. Evid. Based Compl. Altern. Med..

[41] Ousaaid D., Laaroussi H., Bakour M., El Ghouizi A., El Menyiy N., Lyoussi B., El Arabi I. (2022). Effect of a combination of rosa canina fruits and apple cider vinegar against hydrogen peroxide-induced toxicity in experimental animal models. J. Food Qual..

[42] Laaroussi H., Bakour M., Ousaaid D., Aboulghazi A., Ferreira-Santos P., Genisheva Z., Teixeira J.A., Lyoussi B. (2020). Effect of antioxidant-rich propolis and bee pollen extracts against d-glucose induced type 2 diabetes in rats. Food Res. Int..

[43] Lafraxo H., Bakour M., Laaroussi H., El Ghouizi A., Ousaaid D., Aboulghazi A., Lyoussi B. (2021). The synergistic beneficial effect of thyme honey and olive oil against diabetes and its complications induced by alloxan in wistar rats. Evid. Based Compl. Altern. Med..

[44] Bakour M., Soulo N., Hammas N., Fatemi H.E., Aboulghazi A., Taroq A., Abdellaoui A., Al-Waili N., Lyoussi B. (2018). The antioxidant content and protective effect of argan oil and syzygium aromaticum essential oil in hydrogen peroxide-induced biochemical and histological changes. Int. J. Mol. Sci..

[45] Bakour M., Laaroussi H., Ousaaid D., Oumokhtar B., Lyoussi B. (2021). Antioxidant and antibacterial effects of pollen extracts on human multidrug-resistant pathogenic bacteria. J. Food Qual..

[46] Laaroussi H., Bakour M., Ousaaid D., Ferreira-Santos P., Genisheva Z., El Ghouizi A., Aboulghazi A., Teixeira J.A., Lyoussi B. (2021). Protective effect of honey and propolis against gentamicin-induced oxidative stress and hepatorenal damages. Oxid. Med. Cell. Longev..

[47] Arzami A.N., Ho T.M., Mikkonen K.S. (2022). Valorization of cereal by-product hemicelluloses: Fractionation and purity considerations. Food Res. Int..

[48] Ben Jalloul A., Chaar H., Tounsi M.S., Abderrabba M. (2022). Variations in phenolic composition and antioxidant activities of Scabiosa Maritima (*Scabiosa Atropurpurea Sub. Maritima* L.) crude extracts and fractions according to growth stage and plant part. S. Afr. J. Bot..

[49] Solaiman M.M. (2021). Agglutination effect of selected medicinal plant leaf crude extracts on ABO blood group. Am. J. Plant Biol..

[50] El Abdali Y., Allali A., Agour M., Lahkimi A., Eloutassi N., Bouia A. (2021). Phytochemical screening, and in vitro antiradical and immunostimulant potential of *Linum usitatissimum* L. Pharmacologyonline.

[51] Senthilganesh J., Ravichandran S., Durairajan R., BalaSubramaniyan S., Krishnasamy L., Veerappan A., Paramasivam N. (2021). Metal sulfide nanoparticles based phytolectin scaffolds inhibit vulvovaginal candidiasis causing Candida Albicans. J. Clust. Sci..

[52] Cavada B.S., Osterne V.J.S., Oliveira M.V., Pinto-Junior V.R., Silva M.T.L., Bari A.U., Lima L.D., Lossio C.F., Nascimento K.S. (2020). Reviewing mimosoideae lectins: A group of under explored legume lectins. Int. J. Biol. Macromol..

